# Supplementary material for: Anatomical variations in Eurasian wolf (Canis lupus lupus) (Carnivora: Canidae) of the salivary glands: A histological and histochemical investigation
Source: PLoS One. 2025 Jan 10;20(1):e0317102. doi: 10.1371/journal.pone.0317102 (PMC11723621; doi:10.1371/journal.pone.0317102)
Supplement: Attachment — Submitted filename: Response to Reviewers.pdf [file pone.0317102.s002.pdf]

Response to Editor and Reviewers

We would like to thank the Editor and Reviewers for the comments on the paper, as these comments led us to an improvement of the work. The manuscript has been corrected with the recommendations of Editor and Reviewer 2. I`m sending to You answers for these comments. All the changes were signed in red.

Answer for the Comments of Editor:

Comment 1. "Please ensure that your manuscript meets PLOS ONE's style requirements, including those for file naming. The PLOS ONE style templates can be found at

https://journals.plos.org/plosone/s/file?id=wjVg/PLOSOne_formatting_sample_main_body.pdf and

https://journals.plos.org/plosone/s/file?id=ba62/PLOSOne_formatting_sample_title_authors_affiliations.pdf"

Answer: The style of our manuscript was improved and the current version meet the PLOS ONE`s style.

Comment 2. "We note that the grant information you provided in the 'Funding Information' and 'Financial Disclosure' sections do not match. When you resubmit, please ensure that you provide the correct grant numbers for the awards you received for your study in the 'Funding Information' section."

Answer: The Funding information was corrected and unified.

Comment 3. "Thank you for stating the following financial disclosure: "The APC was financed by Warsaw University of Life Sciences. "Please state what role the funders took in the study. If the funders had no role, please state: ""The funders had no role in study design, data collection and analysis, decision to publish, or preparation of the manuscript."" If this statement is not correct you must amend it as needed. Please include this amended Role of Funder statement in your cover letter; we will change the online submission form on your behalf."

Answer: The sentence: "The funders had no role in study design, data collection and analysis, decision to publish, or preparation of the manuscript" was added in the text of the manuscript.

Comment 4. "We note that your Data Availability Statement is currently as follows: All relevant data are within the manuscript and its Supporting Information files. Please confirm at this time whether or not your submission contains all raw data required to replicate the results of your study. Authors must share the "minimal data set" for their submission. PLOS defines the minimal data set to consist of the data required to replicate all study findings reported in the article, as well as related metadata and methods (https://journals.plos.org/plosone/s/data-availability#loc-minimal-data-set-definition)."

Answer: The manuscript contain all relevant data. We added the supplementary data as Supplementary file.

Comment 5. "Please include your tables as part of your main manuscript and remove the individual files. Please note that supplementary tables (should remain/ be uploaded) as separate ""supporting information"" files."

Answer: The tables were included in the text. The individual files were removed.

Comment 6. "Please review your reference list to ensure that it is complete and correct. If you have cited papers that have been retracted, please include the rationale for doing so in the manuscript text, or remove these references and replace them with relevant current references. Any changes to the reference list should be mentioned in the rebuttal letter that accompanies your revised manuscript. If you need to cite a retracted article, indicate the article's retracted status in the References list and also include a citation and full reference for the retraction notice."

Answer: The Reference list was corrected.

Answer for Reviewers:

Reviewer 1

Reviewer #1: "The work provides important data on the morphology of the salivary glands of Eurasian Wolf. The methodology used is appropriate for the proposed objectives and the conclusion is concise with the results obtained, which were described in detail. I would like to highlight the quality of the anatomical images and the photomicrographs."

Answer: Dear Reviewer thank you very much for the opinion on the our manuscript.

Reviewer 2

Reviewer #2: "The work entitled "Anatomical Variations in Eurasian Wolf (*Canis lupus lupus*) (Carnivora Canidae) of the Salivary Glands A Histological and Histochemical Investigation" with ID: PONE-D-24-28995 has been designed and conducted well, with a remarkable

conclusion. However, the authors should address some issues before considering the manuscript for publication (minor revision). The manuscript should be revised for linguistic, grammatical, word spacing, and style errors. The format of the whole manuscript and the reference style must comply with the guidelines of the Journal. Title: good, Abstract: good, Introduction: very good, Material and method: very good.
Results: good However the quality of figures should be increased as much as possible. Macroscopic images were recaptured., Discussion: good."

Answer: Dear Reviewer thank you for the opinion on the our manuscript. The text was revised for linguistic, grammatical, word spacing and style errors. The format of the manuscript complies with the guidelines of the Journal, what was corrected. The quality of all figures was improved. All the changes were marked in the text in red.

We are looking forward to your opinion on the paper.

Sincerely Yours,

Karolina Goździewska-Harłajczuk
